# Survival analysis and obstetric outcomes in patients with early stage ovarian cancer undergoing fertility-sparing surgery

**DOI:** 10.1186/s13048-022-01082-1

**Published:** 2022-12-23

**Authors:** Özer Birge, Mehmet Sait Bakır, Selen Doğan, Hasan Aykut Tuncer, Tayup Simsek

**Affiliations:** 1 Department of Gynecology and obstetrics, Nyala Sudan Turkey Training and Research Hospital, West Alezza District Southern, 63311 Nyala, Darfur Sudan; 2 Department of Gynecology Obstetrics, Division of Gynecologic Oncology, Akdeniz University, Antalya, Turkey

**Keywords:** Conservative, Fertility-sparing, Ovarian Cancer, Staging surgery, Survival

## Abstract

**Objective:**

The aim of the present study is to evaluate the long-term outcomes in patients with early stage ovarian cancer undergoing fertility-sparing surgery.

**Methods:**

The present study performed a retrospective analysis of recurrence, pregnancy and survival of a total of 66 patients who were diagnosed with early stage ovarian cancer (stage I) in XXX Faculty of Medicine Hospital between 2004 and 2019. Of these patients, 16 had undergone fertility-sparing surgery, and the remaining 50 patients had undergone radical surgery.

**Results:**

Of 66 eligible patients, 16 had undergone fertility-sparing surgery, and the remaining 50 patients had undergone radical complete surgery. When demographic and descriptive data are taken into consideration, the mean age was 32.6 ± 6.76 years in patients undergoing fertility-sparing surgery and 54.05 ± 10.8 years in patients undergoing complete surgery, and the difference between the groups was statistically significant (*p* = 0.001). Of patients undergoing fertility-sparing surgery, 11 (16.7%) had stage Ia disease (most common), 5 (7.5%) had stage Ic disease, whereas no patient with stage Ib disease was detected. Of patients undergoing complete radical surgery, 32 (48.5%) had stage Ia disease (most common), 1 (1.5%) had stage Ib disease with bilateral ovarian involvement, and stage Ic was the second most common disease stage. Also, stage Ic3 was the most common disease stage (8 patients, 12.1%) among those with stage Ic disease. The rate of recurrence was 4.5% (3 patients) in patients undergoing fertility-sparing surgery, and recurrences occurred at 37 months, 69 months, and 76 months, respectively. A patient with stage Ic3 disease and endometrioid type tumor who developed recurrence at 37 months died at 130 months. Of patients undergoing complete surgery, ten patients (15.2%) developed recurrence, and there was no significant difference between the two groups in terms of recurrence (*p* = 1.00). At the end of 15-year follow-up period, there was no significant difference between patients undergoing fertility-preserving surgery and those undergoing complete surgery in terms of mortality (*p* = 0.668).

**Conclusion:**

The observation of significant findings in terms of the rate of recurrence and disease-free survival following fertility-sparing surgery in patients with low-risk early stage ovarian cancer suggests that survival is positively affected in early stage ovarian cancer.

## Introduction

Ovarian cancer is the leading cause of death among the other gynecological cancers, and it causes death of approximately 150 thousand people annually in the United States [1, 2]. Ovarian cancer is the second most common cancer after endometrial cancer, particularly in developed countries, insidious growth in the pelvic space and the detection of approximately two-thirds of the patients in the advanced disease stages are the most important causes of poor disease prognosis. The disease is often asymptomatic; a patient has stage III or higher disease stage when the most common symptoms such as abdominal distention and abdominal pain occur [2, 3].

Fertility-sparing approach has gained importance in recent years, particularly in early stage ovarian cancer. Organ-sparing surgery is performed most commonly in this group of patients, because 85% of patients with ovarian cancer have epithelial ovarian tumors [1, 4]. In ovarian cancers, organ-sparing approach can be used in stage 1 borderline tumors, germ-cell tumors, sex cord-stromal tumors, and grade 1 early stage (stage 1 and 2A) epithelial tumors. In ovarian cancers, fertility-sparing surgery can be planned after accurate identification of the disease stage, histological subtype of the tumor, and low-risk status. In fertility-sparing conservative approach, uterus and one of the ovaries are preserved, and wedge biopsy of the contralateral ovary has been recommended by various studies for a long period [1, 4, 5].

Only 10–15% of early stage ovarian cancers are observed in young and fertile women, whereas the majority of the patients are in the peri- or postmenopausal period [6]. A desire for advanced age pregnancy due to postponement of pregnancy in the industrialized countries and the development of assistive reproductive technologies have further increased the importance of fertility-sparing surgery in ovarian cancers.

Although many studies to date have demonstrated favorable effects of fertility-sparing surgery in early stage ovarian cancer in terms of pregnancy and no significant difference has been shown in terms of oncological recurrence when compared to patients undergoing radical surgery, the results of long-term follow-up studies are not sufficient despite some promising results [6–11]. Furthermore, successful pregnancies have been noted after chemotherapy followed by surgery, and similar outcomes have been achieved in terms of recurrence and survival compared to those achieved after radical surgery [12, 13]. Also, there was no significant increase in the rate of abortions and congenital anomalies in these successful pregnancies [12, 13]. In one of the largest studies on fertility-sparing conservative surgery conducted by Ditto et al., a comparison between 70 patients undergoing fertility-sparing surgery and 237 patients undergoing radical surgery revealed similar outcomes and no significant difference was found in terms of recurrence [6].

The present study aims to perform a retrospective review of patients with early stage (stage I) ovarian cancer undergoing fertility-sparing conservative surgery and discuss the recurrence rate, survival, pregnancy rate and other demographic data together with the available literature.

## Material and method

The data of 16 patients who underwent fertility-sparing surgery due to early stage ovarian cancer (stage I) and the data of 50 patients who underwent radical staging surgery in the gynecological oncology clinic of XXX Hospital between 2004 and 2019 were retrospectively analyzed. The study was evaluated by the Akdeniz University Faculty of Medicine Clinical Research Ethics Committee and was approved with a decision number KAEK-716 dated 09.09.2020.

A written detailed informed consent was obtained from all patients for the analysis of their data. The inclusion criteria for patients undergoing fertility-sparing surgery were age being 40 years and under, desire to maintain fertility, the diagnosis of early stage (stage Ia, Ib, Ic) ovarian cancer (no intraabdominal gross disease), intact contralateral ovary on macroscopic or biopsy examination, and regular attendance to control visits. Patients with a borderline disease, patients who did not accept fertility-sparing surgery, and those with advanced stage (stage 2–4) serous ovarian cancer or extra pelvic tumor spread were excluded from the study. The clinical staging of the patients was based on the International Federation of Gynecology and Oncology (FIGO) criteria, and histological subtypes were classified according to the World Health Organization (WHO) guidelines [9, 10]. All patients underwent whole abdominal and thoracic computed tomography (CT) or whole abdominal magnetic resonance imaging (MRI) in the preoperative period. All patients were provided information about the standard of care in early stage epithelial ovarian cancer, which involved total hysterectomy, bilateral salpingo-oophorectomy, bilateral pelvic and paraaortic lymphadenectomy, omentectomy, cytological examination, and peritoneal biopsies, while fertility-sparing conservative surgery involved unilateral salpingo-oophorectomy, unilateral lymphadenectomy, peritoneal biopsy, and cytological examination. Staging was performed during surgery in all patients, and the tumor diagnosis, histological subtype, regional lymph node status, and biopsy specimens collected from all sites were evaluated by expert pathologists in a single pathology center. All high-risk patients were scheduled to receive platinum-based adjuvant chemotherapy after surgery. Patients with stage 1A-1B and grade 1–2 early stage ovarian cancer were regarded as having low risk, while patients with grade 3 and stage 1C or higher disease were regarded as having high risk. It was observed that the patients attended control visits every 3 months in the first 2 years and then every 6 months for 3 years. During the control visits, all patients were evaluated with pelvic examination, tranvaginal or transabdominal ultrasound, serum tumor marker measurement, and radiological assessments. It was found that definitive surgery had been offered to patients who have completed their reproductive phase; however, some of these patients accepted surgery, while others rejected surgery and continued attending control visits. The recurrences that occurred during the follow-up period had been detected by imaging methods and pathological examination, when required.

Statistical analysis was performed using SPSS 23.0 (IBM, USA) software package. The categorical variables were analyzed using a chi-square test or Fisher’s exact test. The D’Agostino-Pearson normality test was used to examine whether the data showed Gaussian distribution. A Student’s t-test was used to evaluate parametric data, and a Mann-Whitney U test was used to evaluate nonparametric data. The disease-free survival (DFS) and overall survival (OS) were calculated using the Kaplan-Meier method, and the two groups were compared using log-rank test. In univariate and multivariate analyses, a *p*-value less than 0.05 was considered statistically significant.

## Results

Of 66 patients who met the study inclusion criteria, 16 had undergone fertility-sparing surgery, and 50 had undergone radical surgery. Descriptive statistics showed that the mean age was 32.6 ± 6.76 years in patients undergoing fertility-sparing surgery and 54.05 ± 10.8 years in patients undergoing radical surgery, showing a significant difference between the two groups (*p* = 0.001) (Table 1).

**Table 1:** Clinical and pathological characteristics of the patients

		Fertility-sparing surgery n:16	Complete surgery n: 50	Total n: 66	*P value*
Age (year)	Mean ± SD	32.6 ± 6.76	54.05 ± 10.8	48.8 ± 13.6	***0.001***
BMI	Median (min-max)	25.7 (21.2–34.3)	26.3 (21.0–37.9)		*0.549*
Preop CA-125	Median (min-max)	52.0 (21.0–432)	61 (4.2–6064)		*0.486*
Histological type	Serous	0 (0%)	12 (18.2%)	12 (18.2%)	*NA*
	Mucinosis	3 (4.5%)	7 (10.6%)	10 (15.2%)	
	Endometrioid	11 (16.7%)	18 (27.3%)	29 (43.9%)	
	Clear	2 (3.0%)	9 (13.6%)	11 (16.7%)	
	Mix (serous + endometrioid)	0 (0%)	4 (6.1%)	4.(6.1%)	
Stage	IA	11 (16.7%)	32 (48.5%)	43 (65.2%)	*NA*
	IB	0 (0%)	1 (1.59%)	1 (1.5%)	
	IC1	2 (3%)	7 (10.6%)	9 (13.6%)	
	IC2	2 (3%)	2 (3%)	4 (6.1%)	
	IC3	1 (1.5%)	8 (12.1%)	9 (13.6%)	
Grade	I	9 (13.6%)	18 (27.3%)	27 (40.9%)	*0.216*
	II	4 (6.1%)	11 (16.7%)	15 (22.7%)	
	III	3 (54.5%)	21 (31.8%)	24 (36.4%)	
Lymph status	Yes	16 (24.2%)	42 (63.6%)	58 (87.9%)	*0.183*
	No	0 (0%)	8 (12.1%)	8 (12.1%)	
Recurrence status	Yes	3 (4.5%)	10 (15.2%)	13 (19.7%)	*1.00*
	No	13 (19.7%)	40 (60.65%)	53 (80.3%)	
Status	Live	15 (22.7%)	43 (65.2%)	58 (87.9%)	*0.668*
	Death	1 (1.5%)	7 (10.6%)	8 (12.1%)	
Adjuvant Chemotherapy	CT+	7 (10.6%)	38 (57.6%)	45 (68.2%)	***0.016***
	CT-	9 (13.6%)	12 (18.2%)	21 (31.8%)	
Follow up	Median (min-max)	93.9 (24.8–180.5)	93.8 (6.9–181.4)		
Pregnancy status	Yes	4 (25%)			
	No	12 (75%)			

When the body mass index (BMI) and preoperative Ca-125 values were evaluated, the parameters that are considered to be a risk factor for gynecological cancers, lower values were observed in patients undergoing fertility-sparing surgery, although the difference between the groups was not statistically significant (*p* = 0.549 and *p* = 0.486, respectively).

In the examination of disease stage in 66 patients with ovarian cancer included in the study, the most common disease stage in patients undergoing fertility-sparing surgery was stage Ia (11 patients, 16.7%), five patients (7.5%) had stage Ic disease, while no patient with stage Ib disease was detected. The most common disease stage in patients undergoing radical surgery was stage Ia (32 patients, 48.5%), one patient (1.5%) had bilateral ovarian involvement (stage Ib), and the second most common disease stage was stage Ic. Also, stage Ic3 was the most common disease stage (8 patients, 12.1%) among patients with stage Ic disease.

When the tumor grade of the patients was examined, Grade I disease was the most common (13.6%) in patients with early stage disease undergoing fertility-sparing surgery which was an important advantage in this particular group, while Grade III disease was the most common (21 patients, 31.8%) in patients undergoing radical surgery, supporting the decision of performing radical surgery in this group of patients. The difference between the groups, however, was not statistically significant (*p* = 0.216).

All patients in the fertility-sparing surgery group (16 patients, 100%) underwent lymphadenectomy, while 42 patients (84%) in the radical surgery group underwent lymphadenectomy; the difference between the groups was not significant (*p* = 0.183).

When the recurrences were evaluated in 66 patients during a 15-year follow-up period, the rate of recurrence was 4.5% (3 patients) in fertility-sparing surgery group, and the recurrences occurred at 37, 69 and 76 months, respectively. A patient with stage Ic3 disease and endometrioid type tumor who developed recurrence at 37 months died at 130 months. Of patients undergoing complete surgery, ten patients (15.2%) developed recurrence, and there was no significant difference between the two groups in terms of recurrence (*p* = 1.00).

During a 15-year follow-up period, no significant difference was observed between fertility-sparing surgery group and radical surgery group in terms of mortality (*p* = 0.668). Seven patients (10.6%) in the fertility-sparing surgery group received six cycles of carboplatin+paclitaxel-based adjuvant chemotherapy. Thirty-eight patients (57.6%) in the radical surgery group received six cycles of carboplatin+paclitaxel-based chemotherapy, and some patients developed peripheral neuropathy associated with chemotherapy. There was a significant difference between the two groups in terms of the receipt of chemotherapy (*p* = 0.016).

The mean duration of follow-up was 93.9 (24.8–180.5) months in the fertility-sparing surgery group and 93.8 (6.9–181.4) months in the radical surgery group.

The disease-free survival was 146 months in the fertility-sparing surgery group and 144 months in the radical surgery group (*p* = 0.724). Similarly, the overall survival was 173 months in the fertility-sparing surgery group and 158 months in the radical surgery group (*p* = 0.387). A comparison between patients undergoing fertility-sparing surgery and those undergoing radical surgery did not show a significant difference in terms of overall survival (OS) and disease-free survival (DFS) (Table 2).

**Table 2:** Impact of surgery type on overall survival (OS) and progression-free survival (PFS) in 66 patients with early stage epithelial ovarian cancer

Surgical treatment	PFS (95% CI), months	*p-Value*	OS (95% CI), months	*p-Value*
Fertility-sparing surgery	146.6 (114.0–179.3)	*0.724*	173.2 (160.1–186.3)	0.387
Complete surgery	144.6 (124.6–164.6)		158.3 (142.8–173.9)	

*CI* Confidence interval

**Fig. 1:**
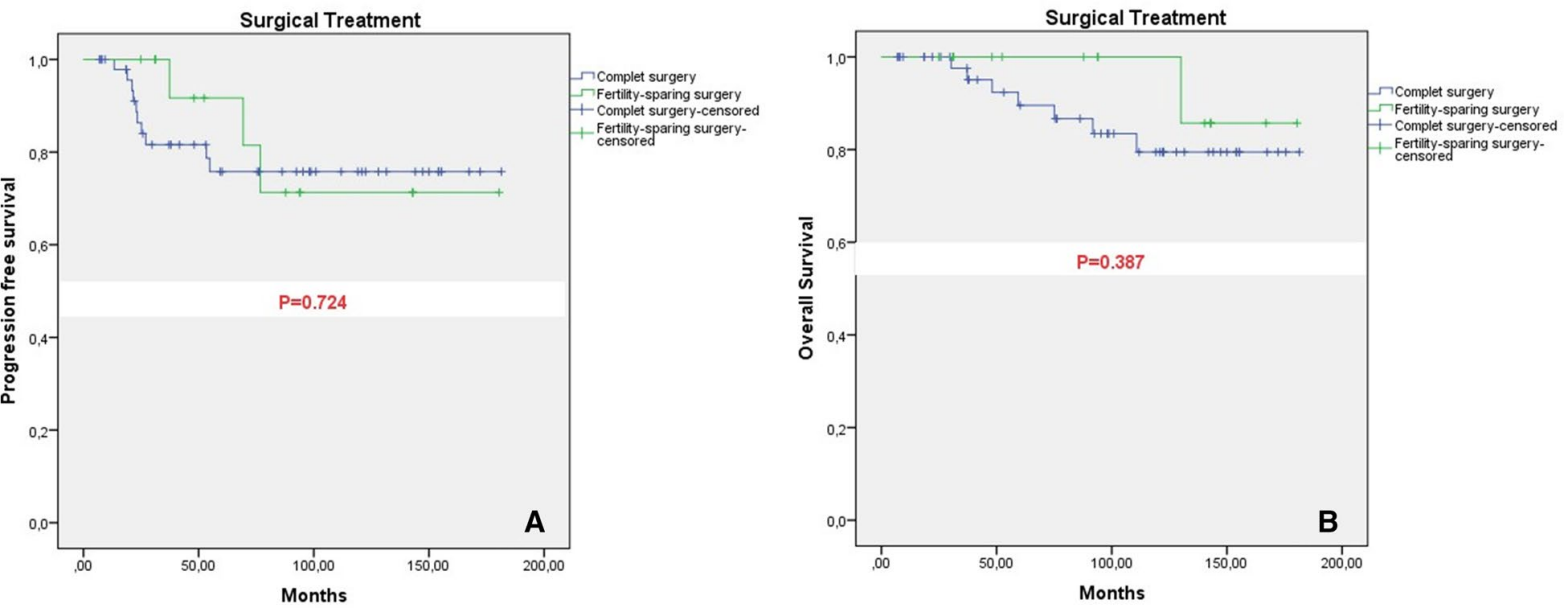
**A** Surgical treatment and PFS, **B** Surgical treatment and OS

Five-year cumulative overall survival was 89.5% in patients with early stage epithelial ovarian cancer undergoing radical surgery and 100% in patients undergoing fertility-sparing surgery; one patient died during the follow-up period after 5 years. Five-year cumulative progression-free survival (PFS) was 75.8% in the radical surgery group and 91.8% in the fertility-sparing surgery group (Fig. 1).

A total of four pregnancies (25%) were observed in 16 patients undergoing fertility-sparing surgery, through in vitro fertilization and embryo transfer in 3 patients and spontaneously in 1 patient (Table 3). Of these pregnancies, three resulted in a term delivery of healthy babies, while one resulted in spontaneous abortion at 7 weeks of gestation. A 34-year-old patient with stage Ic3 endometrioid ovarian cancer and a patient who developed recurrence at 48 months and received chemotherapy are in remission and still on follow-up.

**Table 3:** Clinical characteristics of pregnancy after fertility sparing surgery

Patients name	Age	Stage	Histology	Grade	Type of surgery	Adjuvant chemotherapy	Interval ^a^ (months)	Obstetrics outcome	Status
S.G.	43	Ic1	Clear cell	III	FSS	NO	30	Abortion (7 weeks)	NED
H.E.	34	Ic3	Endometrioid	II	FSS	6x(Paclitaxel+carboplatin)	19	Alive baby	AWD
T.U	30	Ia	Endometrioid	I	FSS	NO	21	Alive baby	NED
N.A	26	Ia	Mucinous	I	FSS	NO	43	Alive baby	NED

*NED* No evidence of disease, ^a^*AWD* Alive with disease, Interval; the time between treatment end and conception

## Discussion

The standard surgical approach to epithelial ovarian cancer involves hysterectomy and bilateral salpingo-oophorectomy, and surgical staging is performed through peritoneal cytological examination, omentectomy, bilateral pelvic and paraaortic lymphadenectomy, and multiple peritoneal biopsies. The purpose of surgical staging is to determine the disease stage and the need for additional therapies [14].

Among non-epithelial ovarian cancers, malignant germ cell and sex cord stromal tumors each constitute 5% of all malignant ovarian tumors. Fertility-sparing surgery is used as the standard treatment for germ cell tumors, especially in women of reproductive age, and these tumors are extremely sensitive to chemotherapy. In addition, sex cord stromal tumors are seen at all ages throughout a woman’s life, but most frequently occur in the menopausal period, and fertility-sparing surgery can be performed in young patients with granulosa cell tumor on histolopathological examination [15].

The removal of ovarian tissue and / or uterus may affect ovarian reserve in the short or long term. Therefore, women who have undergone ovarian surgery may have to use drugs that stimulate ovarian functions in the short or long term in order to achieve pregnancy. However, pregnancies that develop spontaneously without any treatment are also reported [16].

A decline in reproductive functions and hormon levels following fertility-sparing surgery and chemotherapy is the most common concern among young patients who have a desire to conceive. In one of the largest series of patients on this subject reported by Ceppi et al., the examination of reproductive functions in 198 patients between 1980 and 2014 revealed that pregnancy outcomes and endocrine functions remained unaffected following fertility-sparing surgery and chemotherapy, and the rate of premature ovarian insufficiency was low and pregnancy rates were high [17].

Organ-sparing conservative and functional surgical procedures have become more commonly practiced in gynecological cancers. Because preserving the uterus and the ovaries may maintain fertility in patients of reproductive age. The fertility-sparing surgery therefore aims to preserve at least one ovary and/or uterus in women of reproductive age with early stage disease. The fertility-sparing surgery was introduced by Munnel et al. in 1960 to allow young patients with early stage epithelial ovarian cancer to have a child [18]. After the first report in 1960, various case series have been reported on fertility-sparing surgery.

Fertility-sparing surgery was recommended for patients with stage Ia epithelial ovarian cancer and non-clear cell grade 1 and grade 2 tumors by the American College of Obstetricians and Gynecologists (ACOG) in 2007 and the European Society for Medical Oncology (ESMO) in 2008 [19, 20]. The National Comprehensive Cancer Network (NCCN) guidelines recommended the preservation of only the uterus in fertility-sparing surgery in patients with stage Ib or stage Ic disease with bilateral ovarian tumor [14]. Despite these recommendations, many gynecological oncologists still approach fertility-sparing surgery with caution in young patients with a desire to maintain fertility, and direct patients to radical surgery.

Although many large studies recommend that fertility-sparing surgery is not sufficient in terms of oncological outcomes when compared to standard radical surgery in ovarian cancer due to the fact that no clear data can be obtained regarding the status of the remaining ovarian tissue, and that high-risk patients with stage Ic and grade 3 disease must be carefully evaluated for fertility-sparing surgery, some studies reporting on the largest retrospective series to date did not provide evidence for improved oncological outcomes in patients with stage I epithelial ovarian cancer undergoing radical surgery [6, 21, 22].

The present study included 66 patients with stage I epithelial ovarian cancer, and stage Ia was the most common disease stage. Eleven out of 16 patients (68.7%) had stage Ia disease. All patients had epithelial ovarial tumor, the most common subtype was endometrioid tumor both in the fertility-sparing surgery and the radical surgery groups, whereas no serous histology was observed in the fertility-sparing surgery group.

A comprehensive counseling must be provided to the patients regarding the preservation of genital organs during preoperative assessment, particularly to young patients with incidentally-detected early stage epithelial ovarian cancer, and fertility-sparing interventions must be performed and the patients must be given the chance of becoming pregnant soon after the treatment or in the future. It was reported that the rate of pregnancy may be as high as 80% in well-selected young patients, particularly in those with grade I tumor and mucinous histology [8]. Although no data exists regarding as to which patient groups are eligible for fertility-sparing surgery because case-control studies on fertility-sparing surgery cannot be performed on the control group and the patient group due to ethical reasons, it has been reported that the most influential factor on survival is the disease grade determined according to the FIGO [6]. Fertility-sparing surgery can be performed in young patients with low-grade epithelial tumors who have a desire to conceive a child. Thirteen out of 16 patients (82.25%) in the fertility-sparing surgery group had a low-grade tumor in our study, and consistent with the literature, patients with a low-grade tumor have been given a chance to conceive. The mean age was 32.6 ± 6.76 years in the fertility-sparing surgery group and significantly lower than the mean age in the radical surgery group. This finding was consistent with the publications in the literature [9–11]. When the patients were evaluated in terms of histological findings, endometrioid ovarian cancer (68.75%) was the most common histological subtype followed by mucinous histology.

Many studies have a reported pregnancy rates ranging from 35 to 40% in patients undergoing fertility-sparing surgery [9, 10, 21, 23]. In the present study, 4 out of 16 patients (25%) became pregnant, one of which resulted in spontaneous abortion, while three resulted in the delivery of a healthy term baby. Lower pregnancy rates in the present study compared to the reported rates in other studies can be explained by higher mean age in our study, difficulty in accessing assistive reproductive techniques in Turkey, and the reflection of the distress caused by having a cancer at all stages of life in the Turkish community, which has a parochial culture.

In a recent study involving 105 patients, 45 patients with germ cell ovarian tumors requested pregnancy and a pregnancy rate of as high as 93% was reported in the follow-up period. The authors stated that seven patients had received pregnancy treatment persistently and the live birth rate was 86%. Based on their findings, it was emphasized that the outcomes of patients who desire spontaneous pregnancy and those who receive treatment for pregnancy should be evaluated carefully [24].

The place of lymphadenectomy as part of the staging process is controversial in fertility-sparing surgery, and many studies have reported that it has no therapeutic role but has a role in the diagnosis and prognosis. However, one randomized study reported a detection rate of 22% for systematic lymphadenectomy and 9% for lymph node sampling, showing a significant difference (*p* = 0.007) between the two methods [25]. In the present study, all patients in the fertility-sparing surgery group underwent lymphadenectomy (100%), while this rate was lower in the radical surgery group. The reason was that seven patients in the radical surgery group had mucinous histology, and the authors avoided increasing the operation time to reduce morbidity in one patient.

It must be kept in mind that the administration of chemotherapy in patients undergoing optimal fertility-sparing surgery will have no effect on survival but has unfavorable effects on fertility in young patients with a desire to conceive [26]. In the present study, seven out of 16 patients (43.7%) in the fertility-sparing surgery group received chemotherapy, while 38 out of 50 patients (76%) in the radical surgery group received chemotherapy, a rate which is significantly higher than in the fertility-sparing group. The reason for this is the negative effects of chemotherapy on fertility and the presence of patients with early stage disease (stage I) in the study group. In addition, only one patient with stage Ic3 disease among those who conceived in the fertility-sparing surgery group received six cycles of carboplatin+paclitaxel chemotherapy. This patient had conceived a child 19 months after the cessation of cancer therapy. This may be explained by the young age of the patient and the effects of chemotherapy on ovarian reserves being reversible.

In 2016, a study by Fruscio et al. involving patients with epithelial ovarian cancer compared 242 patients undergoing fertility-sparing surgery with 789 patients undergoing radical surgery. The authors reported similar survival rates between the two groups during a follow-up period of 11 years [27].

In the latest retrospective series of 34 patients undergoing fertility-sparing surgery in a study by Bogani et al. in 2020, 17 patients (50%) were in the low risk group with stage Ia, Ib and grade I-II disease. The analysis of the results showed that the outcomes of conservative approach were similar between patients with advanced-stage and high-grade disease, and patients with stage I and grade I-II disease, and the survival outcomes did not significantly differ between the groups [28]. When the outcomes of fertility-sparing surgery was analyzed in the high-risk group, the rate of recurrence was found to be lower in patients undergoing radical surgery. However, retrospective study design and small sample size were reported as the limitations of their study [28]. In the present study, the number of patients with stage Ia and grade I-II disease was higher, a finding consistent with that in the literature. The inclusion of as low as 16 patients in the fertility-sparing surgery group despite the total number of patients being 66 in the study, and retrospective study design can be regarded as the limitations of the present study. On the other hand, the present study is valuable due to long follow-up period of 15 years despite small sample size.

In an analysis of mortality rates, Melamed et al. reviewed the American National Cancer Database (NCDB) between 2004 and 2012, and identified 1726 patients aged under 40 years with stage Ia and unilateral stage Ic disease. Of these patients, 825 (47.8%) had undergone fertility-sparing surgery, and young age, living in urban areas, and serous and mucinous histology were identified as the most important factors associated with a fertility-sparing approach. Furthermore, when radical surgery was compared to conservative approach in terms of mortality in a 63-month period, 30 patients died in the conservative group and 37 patients died in the radical surgery group, showing no significant difference between the groups [29]. In the present study, one patient died in the fertility-sparing group, and 7 patients died in the radical surgery group. Consistent with the literature, a comparison between the two groups showed no significant difference in terms of progression-free survival and overall survival during a 15-year follow-up period.

Recently, minimally invasive surgical methods have been increasing in the treatment of early-stage epithelial ovarian cancer. However, the impact of minimally invasive surgical methods versus laparotomy on survival remains the most important concern. In one of the studies that reduced this anxiety; Multicenter retrospective 254 early stage epithelial ovarian cancers were treated with minimally invasive methods (188 with laparoscopic surgical staging, 66 with robotic surgical staging). After a median follow-up of 61 months (the longest in the literature), the 5-year progression free survival (PFS) and overall survival rates were 84 and 93.8%, respectively. This study showed that minimally invasive treatment modalities have valuable therapeutic contributions in early stage epithelial ovarian cancer in selected patients [30].

In another Italian study investigating whether there is a difference between minimally invasive methods (laparoscopy versus robotic) in epithelial early stage ovarian cancer, 34 patients were treated with robotic laparoscopy and 62 patients were treated with laparoscopy. It was shown that there was no difference between the two groups in terms of both the number of pelvic paraaortic lymph nodes and postoperative complications. This study, which had a shorter operation time in the patient group treated with the robotic method, revealed that minimally invasive surgery is safe and feasible. In addition, in this study, 6 patients underwent fertility-sparing surgery (2 robotic, 4 laparoscopy) [31].

The present study naturally has some limitations; retrospective study design may have caused biases in the study results. Currently, it does not seem feasible to conduct randomized clinical studies due to ethical and legal concerns in patients with early stage epithelial ovarian cancer who are scheduled for fertility-sparing surgery.

## Conclusion

Fertility-sparing surgery can be safely offered to patients with epithelial ovarian cancer who have a desire to conceive. However, after carefully weighing out the advantages and the disadvantages of fertility-sparing surgery in early stage (stage I) ovarian cancer, unfavorable consequences of disease progression must be kept in mind despite the advantages conferred by preserving the genital organs and giving the patient and her relatives the opportunity of conceiving a child.

## Data Availability

The datasets used and/or analysed during the current study are available from the corresponding author on reasonable request.
